# Timing of veno-arterial extracorporeal membrane oxygenation in cardiogenic shock: A systematic review and meta-analysis

**DOI:** 10.1016/j.jointm.2026.01.005

**Published:** 2026-02-19

**Authors:** Xiang-kun Yuan, Rui Shi, Lu-ming Zhang, Xiang-jie Duan, Li-jun Li, Hai-yan Yin, Wan-jie Gu, Xavier Monnet

**Affiliations:** 1 Department of Intensive Care Unit, The First Affiliated Hospital of Jinan University, Guangzhou, Guangdong, China; 2 Department of Critical Care Medicine, The First Affiliated Hospital of Sun Yat-Sen University, Guangzhou, Guangdong, China; 3 Service de Médecine Intensive-Réanimation, AP-HP, Hôpital de Bicêtre, DMU 4 CORREVE, Université Paris-Saclay, Le Kremlin-Bicêtre, Paris, France

**Keywords:** Cardiogenic shock, Mechanical circulatory support, VA-ECMO, Timing, Mortality

## Abstract

**Background:**

Although the use of veno-arterial extracorporeal membrane oxygenation (VA-ECMO) in cardiogenic shock has increased, the optimal timing for its initiation is still unclear. This study aimed to evaluate whether early initiation of VA-ECMO in patients with cardiogenic shock is associated with improved outcomes compared with delayed initiation.

**Methods:**

This systematic review and meta-analysis was prospectively registered in PROSPERO (CRD42025635381). We searched PubMed, Embase, and the Cochrane Library from their inception to December 11, 2024, to identify published studies that compared early *vs.* delayed initiation of VA-ECMO in cardiogenic shock and that provided clear timing definitions based on each included study. The primary outcome was short-term mortality. Secondary outcomes included complications (neurological, bleeding, and ischemic) and long-term mortality. Sensitivity analyses were conducted using adjusted estimates, alternative timing definitions (prerevascularization or approximate 2 h after shock diagnosis), and exclusion of the largest contributing study. Subgroup analyses were conducted to explore heterogeneity by VA-ECMO initiation definition and cardiogenic shock etiology. Odds ratios (ORs) with 95% confidence intervals (CIs) were pooled using a fixed- or random-effects model according to heterogeneity.

**Results:**

Eight observational studies involving 10,451 patients were included. Early VA-ECMO initiation was associated with reduced short-term mortality compared to delayed initiation (OR=0.74, 95% CI: 0.58 to 0.96, *P*=0.02). No significant differences were found between the two groups for neurological (OR=0.67, 95% CI: 0.34 to 1.33, *P*=0.25), bleeding (OR=0.83, 95% CI 0.56 to 1.23, *P*=0.36), or ischemic (OR=0.87, 95% CI: 0.32 to 2.41, *P*=0.79) complications and long to term mortality (OR=0.63, 95% CI: 0.39 to 1.02, *P*=0.06). Sensitivity analyses obtained consistent results: adjusted estimates (OR=0.69, 95% CI: 0.53 to 0.89, *P* <0.01), pre to revascularization VA-ECMO initiation (OR=0.40, 95% CI: 0.23 to 0.68, *P* <0.01), and initiation approximately 2 h after shock diagnosis (OR=0.69, 95% CI: 0.49 to 0.97, *P*=0.03). Subgroup analyses suggested a potential association between early VA-ECMO initiation and reduced short-term mortality in studies using a temporal definition (OR=0.87, 95% CI: 0.80 to 0.95, *P* <0.01).

**Conclusions:**

Early initiation of VA-ECMO in patients with cardiogenic shock appears to be associated with reduced short-term mortality without an apparent increase in neurological, bleeding, or ischemic complications. As all included studies were observational, randomized controlled trials are needed to determine the optimal timing and patient selection for VA-ECMO initiation.

## Introduction

Cardiogenic shock is a life-threatening condition characterized by acute pump failure due to primary cardiac disorders, leading to a rapid decline in cardiac output.^[^^1^^,^^2^^]^ Given its time-sensitive nature, immediate interventions such as inotropes and vasopressors are commonly administered to stabilize hemodynamics and maintain sufficient cardiac output, ensuring proper organ perfusion.^[^^3^^,^^4^^]^ However, conventional pharmacological therapies have several limitations, including metabolic disorders, arrhythmic events, and increased myocardial oxygen demand,^[^[5], [6], [7]^]^ and they may have limited efficacy. These challenges have driven the increasing use of mechanical circulatory support devices, which offer direct hemodynamic support as short-term therapies. Devices such as the intra-aortic balloon pump, Impella, and venoarterial extracorporeal membrane oxygenation (VA-ECMO) have become essential tools in managing cardiogenic shock.^[^^3^^,^^7^^]^ Unlike other devices, VA-ECMO provides both left and right ventricular support through its circulatory assistance mechanism.^[^^8^^]^

With technological advancements, VA-ECMO has become a widely utilized approach in the management of cardiogenic shock.^[^^9^^,^^10^^]^ A meta-analysis reported improved 30-day survival with VA-ECMO support in patients with cardiogenic shock or cardiac arrest,^[^^11^^]^ while a randomized controlled trial found no significant mortality benefit from VA-ECMO support.^[^^12^^]^ Despite this, the positive hemodynamic improvements observed following VA-ECMO highlight the need for further investigation into its optimal application.^[^^3^^]^ A critical gap in knowledge remains regarding the optimal timing for VA-ECMO initiation – a key factor influencing end-organ perfusion, multiple organ dysfunction syndrome, and complications, all of which significantly affect clinical outcomes.^[^^13^^]^

The available evidence on early VA-ECMO initiation is conflicting. Although some studies suggest early initiation was associated with reduced mortality,^[^^14^^,^^15^^]^ others failed to show significant benefits in terms of survival.^[^^16^^,^^17^^]^ Due to potential complications, VA-ECMO is often reserved for cases of refractory cardiogenic shock that do not respond to conventional treatments.^[^^18^^]^ Most existing evidence comes from studies focusing on cardiogenic shock secondary to acute myocardial infarction (AMI),^[^^19^^]^ with an emphasis on the timing of VA-ECMO initiation in relation to revascularization procedures.^[^^14^^,^^20^^,^^21^^]^ However, given the diverse etiologies of cardiogenic shock, it remains uncertain whether there is a universally optimal timing for VA-ECMO initiation across different patient populations.

To address this gap, we performed a meta-analysis of existing studies examining the timing of VA-ECMO initiation in patients with cardiogenic shock. Our primary aim was to determine whether early initiation of VA-ECMO is associated with reduced short-term mortality compared with delayed initiation. Secondary objectives included comparing complication rates and long-term mortality between early and delayed VA-ECMO initiation.

## Methods

This study was prospectively registered in PROSPERO (CRD42025635381) and reported following the meta-analysis of observational studies in epidemiology reporting guidelines.^[^^22^^]^

### Literature search and study selection

Two reviewers (R.S. and X.K.Y.) independently searched PubMed, Embase, and the Cochrane Library using the terms: extracorporeal membrane oxygenation, cardiogenic shock, and timing. The full search strategy for each database is outlined (Supplementary Table S1). Additionally, a manual search was conducted on the reference lists of eligible studies and relevant previous reviews. No further restrictions were applied during the search, which was last updated on December 11, 2024.

The reviewers (R.S. and X.K.Y.) independently screened the search results, removed duplicates, and assessed titles and abstracts for eligibility. Full texts of the selected studies were then retrieved and assessed to identify eligible studies. In cases of disagreement, a third reviewer (H.Y.Y.) was consulted to resolve any discrepancies.

### Eligibility criteria

Studies were included if they met the following criteria: (1) Population: adult patients with cardiogenic shock undergoing VA-ECMO; (2) Exposure and comparison: eligible studies were required to clearly define early and delayed VA-ECMO initiation. The exposure group comprised patients receiving early VA-ECMO support, while the comparison group comprised those with delayed VA-ECMO support; (3) Outcomes: available data on mortality, including raw event counts, odds ratios (ORs), hazard ratios (HRs), or Kaplan–Meier survival curves; and (4) Design: randomized controlled trials and cohort studies. Case reports, reviews, commentaries, letters, editorials, meeting abstracts, and guidelines were excluded.

For classification, patients were divided into early and delayed groups based on the definitions in each included study, without specific restrictions on the timing of VA-ECMO initiation. Additionally, the types of cardiogenic shock were not limited, allowing inclusion of causes related to AMI, postcardiotomy shock, or others. The primary outcome was short-term mortality, defined as 30-day mortality. If data for this time point were unavailable, we used in-hospital mortality or mortality at the nearest available time point. Secondary outcomes included neurological complications, bleeding complications, ischemia complications, and long-term mortality. The criteria for defining these outcomes and their evaluation timelines were adopted from the original reports (Supplementary Table S2).

### Data collection and quality assessment

Two reviewers (R.S. and X.K.Y.) independently screened the full texts and supplementary materials, extracting the following information: first author, publication year, country, number of patients, type of cardiogenic shock, and study design.

Additionally, the data collection sheet captured study-specific definitions of VA-ECMO initiation timing, including how early and delayed initiation were defined in each study, as well as outcomes and adjusted covariates. Data collection was conducted independently by two reviewers, with discrepancies resolved through discussion with a third reviewer (H.Y.Y.).

The quality of the included studies was assessed using the Newcastle-Ottawa Quality Assessment Scale.^[^^23^^]^ Studies with a score of 3 or below were considered low quality, those with a score of 4–6 were rated as moderate quality, and studies with a score of 7 or higher were classified as high quality.

### Statistical analysis

Dichotomous data were analyzed using ORs with 95% confidence intervals (CIs), and pooled using fixed- or random-effects model to account for potential heterogeneity. Mortality was reported as the number of deaths and the total number of participants in both the early and delayed groups. If study groups were divided into more than two categories, we combined them according to the definitions of early and delayed groups provided by the majority of the included studies. Heterogeneity between studies was assessed using Cochrane’s *Q* test (*P* <0.10) and the *I*^2^ statistic (with an *I*^2^ value greater than 50% considered indicative of significant statistical heterogeneity). The forest plot was used to visually present the overall effect and the weight of each study. A two-tailed *P* value of less than 0.05 was considered statistically significant in all analyses. Publication bias was assessed using funnel plots and the Egger test. All statistical analyses were conducted using RevMan version 5.4 (The Cochrane Collaboration) and R software version 4.3.1 (R Foundation for Statistical Computing, Vienna, Austria).

### Sensitivity and subgroup analyses

Sensitivity analyses were performed to evaluate the robustness of the results by restricting the analysis to studies reporting adjusted estimates, using different definitions of early VA-ECMO initiation (prerevascularization or approximate 2 h after shock diagnosis), and excluding the largest contributing study. Subgroup analyses were conducted based on two dimensions: (1) the definition of early VA-ECMO initiation, comparing studies using procedural definitions with those using temporal definitions; and (2) the etiology of shock, including AMI, postcardiotomy, and mixed populations.

## Results

### Study selection and quality assessment

**Figure 1:**
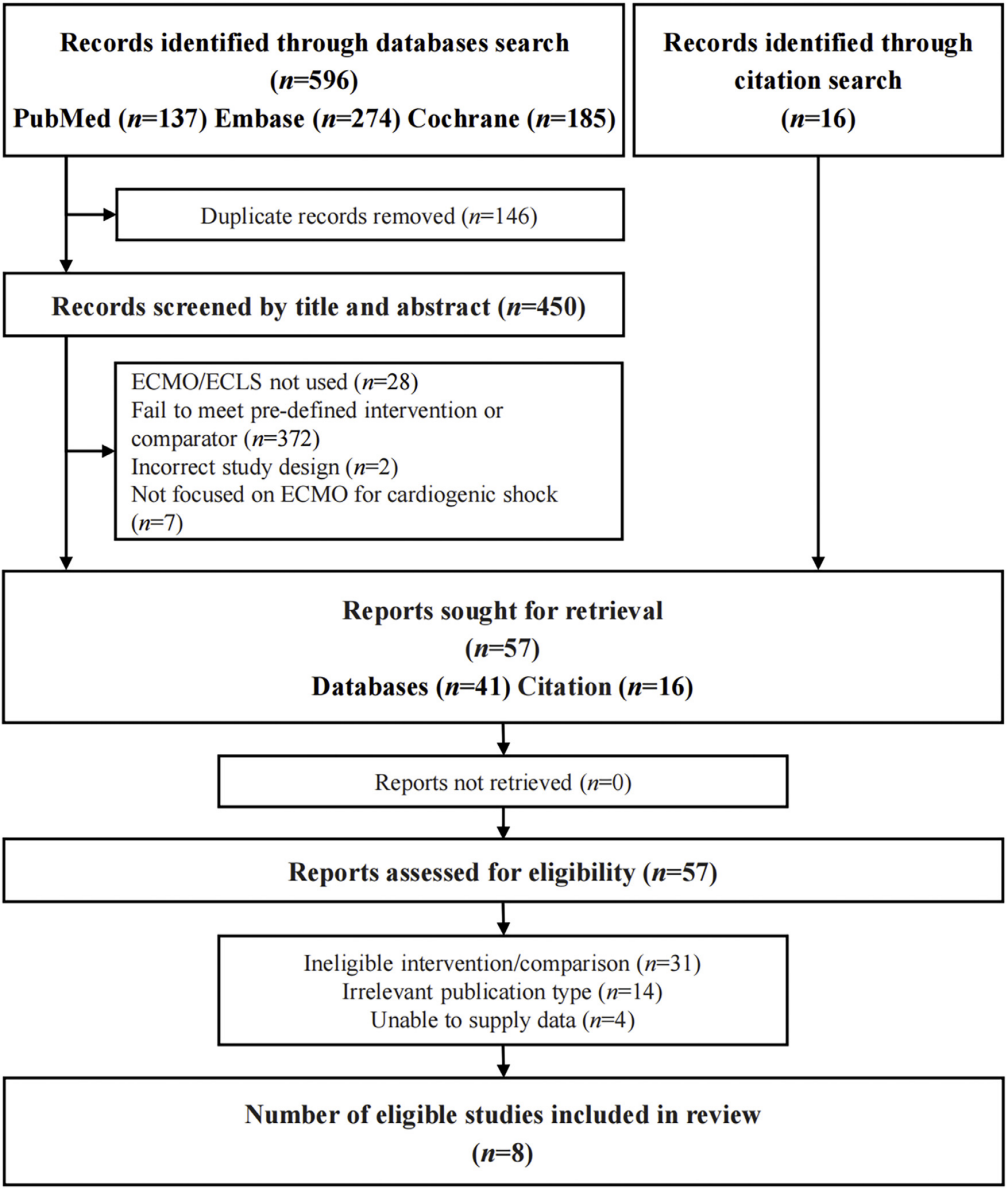
Study flowchart.

The study selection process is outlined in Figure 1. A total of 146 duplicate records were removed after searching PubMed, Embase, and the Cochrane Library. After reviewing 450 articles, full-text articles were retrieved and screened based on the eligibility criteria. Additionally, 16 articles were manually identified through reference lists. Ultimately, eight studies were included in the analysis.

Quality assessment was carried out using the Newcastle-Ottawa Scale, and the results are presented (Supplementary Table S3). The average overall score across the included studies was 7, with scores ranging from 5 to 9.

### Study characteristics

The characteristics of the included studies are shown in Table 1. The final analysis included eight studies published between 2018 and 2024, performed in six countries. Of these, four studies focused on AMI; two on general cardiogenic shock; one on postcardiotomy cardiogenic shock, and one on refractory cardiogenic shock. The diagnostic criteria for studies enrolling patients with mixed etiologies of cardiogenic shock are presented (Supplementary Table S4). The early and delayed VA-ECMO groups were classified using procedural^[^^14^^,^^17^^,^^20^^,^^21^^]^ or temporal definitions.^[^^15^^,^[24], [25], [26]^]^ In the temporal definition subgroup, three of the four studies reported more than two timing categories,^[^[24], [25], [26]^]^ and the regrouping into early and delayed VA-ECMO initiation is presented (Supplementary Table S5). A comprehensive summary of ECMO initiation criteria for each included study is provided (Supplementary Table S6). The major endpoint for short-term outcomes was 30-day mortality.^[^^14^^,^^16^^,^^20^^,^^25^^,^^26^^]^ For studies reporting in-hospital mortality,^[^^15^^,^^21^^,^^24^^]^ the corresponding lengths of hospital stay are presented (Supplementary Table S7). Adjusted estimates and covariates were reported within four studies.^[^^14^^,^^15^^,^^21^^,^^25^^]^

**Table 1:** Characteristics of included studies.

Study	Publication year	Study design	Number of patients	Country	Etiology of cardiogenic shock	Timing of ECMO		Outcomes	Patients
						Early group (*n*=6945)	Delayed group (*n*=3506)		
Benseghir et al.^[^^24^^]^	2021	Retrospective cohort	114	France	Cardiac surgery	≤24 h after aortic unclamping	24–48 h after aortic unclamping	In-hospital mortality	Postcardiotomy refractory cardiogenic shock
Choi et al.^[^^21^^]^	2020	Prospective cohort	147	Korea	AMI	Pre-revascularization	Postrevascularization	In-hospital mortality	AMI complicated by refractory cardiogenic shock
Huang et al.^[^^20^^]^	2018	Prospective cohort	46	China	AMI	Pre-PCI	Post-PCI	30-day mortality	ST-segment elevation myocardial infarction and refractory cardiogenic shock
Jentzer et al.^[^^15^^]^	2024	Retrospective cohort	8619	USA	Mixed	≤24 h from hospital admission	>24 h from hospital admission	In-hospital mortality	Cardiogenic shock
Kim et al.^[^^14^^]^	2021	Prospective cohort	184	Korea	AMI	Pre-PCI	Post-PCI	30-day mortality	AMI complicated by profound cardiogenic shock
Lee et al.^[^^25^^]^	2021	Prospective cohort	362	Korea	Mixed	≤2.2 h from shock diagnosis	>2.2 h from shock diagnosis	30-day mortality	Refractory cardiogenic shock
Pozzi et al.^[^^17^^]^	2023	Retrospective cohort	649	France	AMI	On the day of PCI	Beyond the day of PCI	90-day mortality	AMI complicated by cardiogenic shock
Sundermeyer et al.^[^^26^^]^	2024	Retrospective cohort	330	Germany, Italy, USA	Mixed	<2 h from shock diagnosis	≥2 h from shock diagnosis	30-day mortality	Cardiogenic shock

AMI: Acute myocardial infarction; ECMO: Extracorporeal membrane oxygenation; PCI: Percutaneous coronary intervention.

### Primary outcome

**Figure 2:**
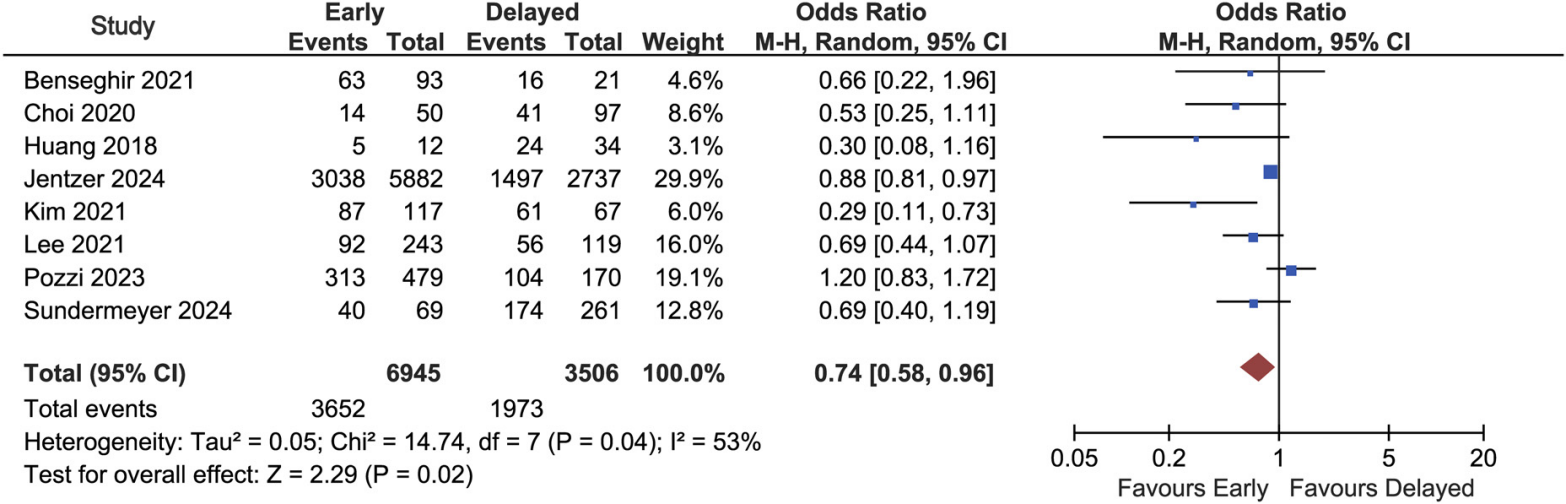
Forest plot for short-term mortality comparing early *vs.* delayed VA-ECMO initiation in patients with cardiogenic shock. CI: Confidence interval; VA-ECMO: Venoarterial extracorporeal membrane oxygenation.

Eight observational studies involving 10,451 participants (6945 in the early VA-ECMO initiation group *vs.* 3506 in the delayed group) were included in the meta-analysis, with four using 30-day mortality, three using in-hospital mortality, and one using 90-day mortality as the endpoint. Early VA-ECMO initiation was associated with reduced risk of short-term mortality in patients with cardiogenic shock compared with delayed VA-ECMO initiation (OR=0.74, 95% CI: 0.58 to 0.96, *P*=0.02, Figure 2). Moderate heterogeneity was observed (*I*^2^=53%, *P*=0.04, Figure 2).

### Secondary outcomes

**Figure 3:**
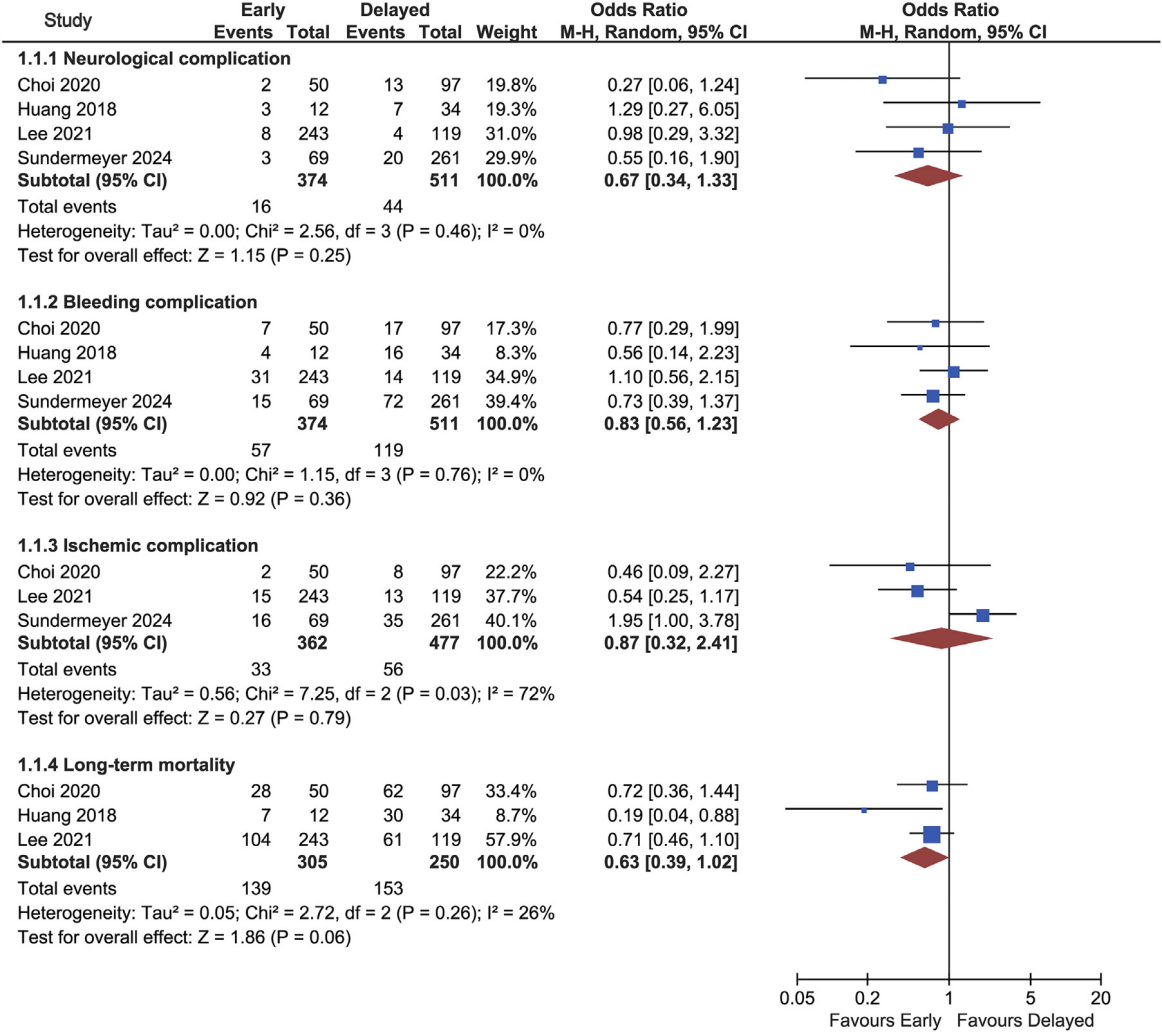
Forest plot for VA-ECMO-related complications (neurological, bleeding, and ischemic) and long-term mortality. CI: Confidence interval; VA-ECMO: Venoarterial extracorporeal membrane oxygenation.

Secondary outcomes included three VA-ECMO-related complications and long-term mortality (Figure 3). No significant differences were observed between early and delayed initiation in neurological (OR=0.67, 95% CI: 0.34 to 1.33, *P*=0.25, *I*^2^=0%), bleeding (OR=0.83, 95% CI: 0.56 to 1.23, *P*=0.36, *I*^2^=0%), or ischemic complications (OR=0.87, 95% CI: 0.32 to 2.41, *P*=0.79, *I*^2^=72%). Three studies reported long-term mortality, and pooled analysis showed that early VA-ECMO initiation was not significantly associated with improved long-term mortality compared with delayed initiation (OR=0.63, 95% CI: 0.39 to 1.02, *P*=0.06, *I*^2^=26%).

### Sensitivity analyses

**Figure 4:**
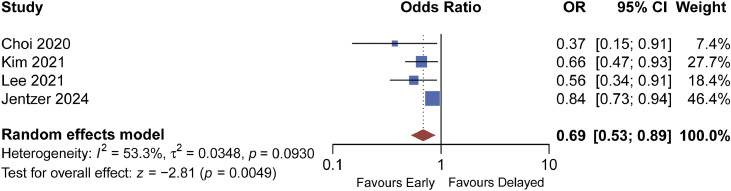
Forest plot of studies reporting adjusted estimates for short-term mortality. CI: Confidence interval; VA-ECMO: Venoarterial extracorporeal membrane oxygenation.

**Figure 5:**
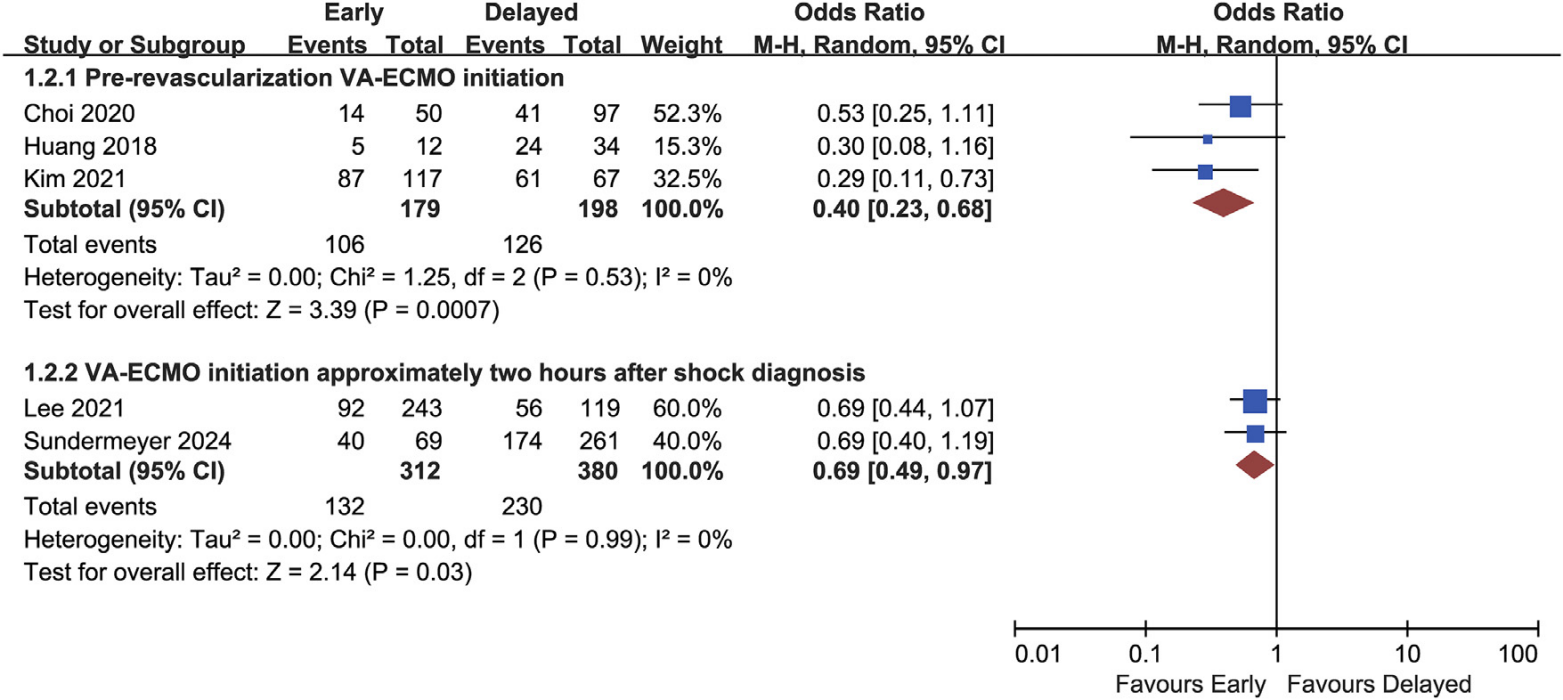
Forest plot for short-term mortality using alternative timing definitions (prerevascularization and approximately 2 h after shock diagnosis). CI: Confidence interval; VA-ECMO: Venoarterial extracorporeal membrane oxygenation.

Sensitivity analyses were conducted to assess the robustness of the primary findings. The results of pooling adjusted estimates indicated that early VA-ECMO initiation was associated with reduced short-term mortality compared with delayed initiation (OR=0.69, 95% CI: 0.53 to 0.89, *P* <0.01, *I*^2^=53.3%, Figure 4). Adjusted estimates and covariates from each study are presented (Supplementary Table S8). When classified by procedural sequence, pre-revascularization VA-ECMO initiation was associated with a lower risk of mortality compared with post-revascularization initiation (OR=0.40, 95% CI: 0.23 to 0.68, *P* <0.01, *I*^2^=0%, Figure 5). Similarly, in studies using temporal thresholds, VA-ECMO initiation approximately 2 h after cardiogenic shock diagnosis was associated with reduced mortality relative to delayed initiation (OR=0.69, 95% CI: 0.49 to 0.97, *P*=0.03, *I*^2^=0%, Figure 5). Exclusion of the largest contributing study (weight=29.9%) yielded results consistent with the primary analysis (OR=0.65, 95% CI: 0.45 to 0.95, *P*=0.02, Supplementary Figure S1).

### Subgroup analyses

Subgroup analyses were performed to explore sources of heterogeneity for the primary outcome. When stratified by definitions of VA-ECMO initiation, early initiation was associated with reduced short-term mortality in studies using temporal definitions (OR=0.87, 95% CI: 0.80 to 0.95, *P* <0.01, *I*^2^=0%, Supplementary Figure S2), while no significant association was observed in studies using procedural definitions (OR=0.54, 95% CI: 0.25 to 1.19, *P*=0.13, *I*^2^=76%, Supplementary Figure 2). Subgroup analyses by cardiogenic shock etiology (AMI, postcardiotomy, and mixed) did not show a consistent benefit of early initiation across etiologies (Supplementary Figure S3).

### Publication bias

No significant publication bias was detected by either the funnel plot or the Egger test (*P*=0.082, Supplementary Figure S4).

## Discussion

This systematic review and meta-analysis of observational studies suggests a potential association between early VA-ECMO initiation and lower short-term mortality in patients with cardiogenic shock, without evidence of an increased risk of VA-ECMO-relevant complications. However, no statistically significant association was observed with long-term mortality.

### Timing and patients

Because high-level evidence is insufficient, the optimal timing for VA-ECMO initiation in cardiogenic shock remains uncertain.^[^^1^^,^^3^^]^ The neurohormonal responses caused by decreased cardiac output and systemic hypoperfusion in cardiogenic shock can increase circulatory burden and exacerbate cardiac dysfunction, thereby forming a vicious cycle.^[^^13^^]^ Additionally, hemodynamic collapse-induced microcirculatory impairment is associated with 30-day mortality.^[^^27^^]^ Early VA-ECMO initiation may improve outcomes by interrupting this cascade through rapid restoration of end-organ perfusion and preventing subsequent circulatory system disorders.^[^^28^^]^ Sensitivity analyses consistently indicated the benefit of early VA-ECMO initiation, which is aligned with the primary outcome.

A key concern regarding pre-revascularization VA-ECMO initiation is its potential impact on door-to-balloon (D2B) time. As D2B time is a critical determinant of outcomes in patients with AMI, clinical experts consistently strive to minimize this interval.^[^^29^^]^ Paradoxically, although D2B times have progressively shortened over time, this trend has not translated into measurable survival benefit.^[^^30^^]^ Previous studies have shown that VA-ECMO initiation before percutaneous coronary intervention does not result in a statistically significant prolongation of D2B time.^[^^20^^,^^29^^]^ Existing evidence also suggests that early VA-ECMO initiation may improve short- and long-term survival, particularly in ST-segment elevation myocardial infarction patients with cardiogenic shock.^[^^20^^,^^31^^,^^32^^]^ Our sensitivity analysis corroborated these findings, showing a consistent reduction in mortality compared with post-revascularization VA-ECMO initiation.

Nevertheless, heterogeneity persisted in subgroup analyses, particularly within the procedural-definition and AMI subgroups. This may partly reflect differences in the operationalization of early *vs.* delayed VA-ECMO initiation, as well as variations in shock etiology. Notably, both subgroup analyses were informed by the same four studies,^[^^14^^,^^17^^,^^20^^,^^21^^]^ although these studies differed in how the timing of VA-ECMO initiation was operationalized. Most studies classified early and delayed initiation according to pre- or postrevascularization,^[^^14^^,^^20^^,^^21^^]^ whereas Pozzi et al.^[^^17^^]^ operationalized timing based on whether VA-ECMO was initiated on or beyond the day of percutaneous coronary intervention. These distinct procedural thresholds may reflect different clinical decision points and contribute to between-study inconsistency. Importantly, when analyses were restricted to studies using a pre- *vs.* post-revascularization framework, heterogeneity was eliminated (*I*^2^=0%), and prerevascularization VA-ECMO initiation was associated with a significantly lower mortality risk.

Although our study suggested a potential short-term survival benefit from early VA-ECMO initiation, the findings of the randomized controlled trial ECMO—CS should be noted. That trial concluded that immediate VA-ECMO initiation did not improve clinical outcomes compared with the early conservative therapy group.^[^^18^^]^ These differences highlight the need to further examine factors that may explain the discrepancy between observational and randomized evidence. One key factor is selection bias: The studies included in this meta-analysis focused on patients who had already received VA-ECMO support, meaning clinicians had likely selected those perceived to have a better prognosis based on their clinical judgment.^[^[14], [15], [16], [17]^,^^20^^,^^21^^,^[24], [25], [26]^]^ In contrast, the ECMO—CS trial minimized this bias by randomizing patients with rapidly deteriorating or severe cardiogenic shock to evaluate routine VA-ECMO use.^[^^18^^]^ Furthermore, the contrast in the ECMO—CS trial was diluted because a considerable proportion of patients (39%) in the conservative therapy group ultimately received VA-ECMO as rescue therapy. Consequently, the ECMO—CS trial addresses the question of routine early initiation *vs.* a judicious rescue strategy, an issue that our meta-analysis of observational studies cannot definitively answer. These findings indicate that uncertainty remains regarding the optimal timing of VA-ECMO initiation.

In light of the available evidence, the latest expert consensus from the American College of Cardiology strongly discourages routine use of temporary Mechanical Circulatory Support (tMCS) in all patients with cardiogenic shock,^[^^33^^]^ but the writing group also notes that delays in tMCS initiation may lead to adverse outcomes, including multiorgan failure and death, in appropriate candidates.^[^^34^^]^ Therefore, despite reaching conflicting conclusions, this meta-analysis and the ECMO—CS randomized controlled trial ultimately point to a core issue raised by current consensus: How to accurately identify patients most likely to benefit from early ECMO intervention.^[^^33^^,^^35^^]^

### Shock stage and VA-ECMO initiation

Against this backdrop, the Society for Cardiovascular Angiography and Interventions (SCAI) shock stage classification offers an easy and rapid framework for assessing shock severity.^[^^36^^,^^37^^]^ In addition, existing data indicate that mortality in cardiogenic shock increases significantly with higher SCAI shock stage.^[^^38^^]^

Only two observational studies included in this meta-analysis reported SCAI shock stage distributions and reached different conclusions.^[^^15^^,^^25^^]^ One study included predominantly patients in stages B–D, with only 1.6% in stage E, and found that early initiation was associated with lower in-hospital mortality.^[^^15^^]^ In contrast, the other study included approximately 71% of patients who were in SCAI shock stage E – indicative of actual or impending circulatory collapse – and observed no significant difference in outcomes between early and delayed initiation.^[^^25^^]^ Similarly, the ECMO—CS trial, which found no survival benefit of immediate ECMO initiation after shock diagnosis, enrolled exclusively patients with cardiogenic shock in SCAI shock stages D and E.^[^^18^^]^

These findings collectively suggest that the shock stage at the time of VA-ECMO initiation may provide a new perspective for understanding which patients are most likely to benefit from early intervention. In other words, the definition of early VA-ECMO initiation could shift from reliance on the time point of shock diagnosis or revascularization to a reference axis based on the SCAI shock classification, which enables dynamic assessment of patients with cardiogenic shock and timely identification of appropriate candidates.^[^^37^^]^

### Safety and efficacy

Only three studies reported long-term mortality.^[^^20^^,^^21^^,^^25^^]^ Although the point estimate suggested a possible trend toward reduced long-term mortality with early VA-ECMO initiation, no statistically significant benefit was observed in the pooled analysis, and the wide CI indicates considerable uncertainty. The multicenter randomized ECMO—CS trial found no overall improvement in 1-year survival associated with immediate VA-ECMO initiation.^[^^39^^]^ However, a *post hoc* subgroup analysis suggested a potential benefit in patients with baseline mean arterial pressure <60 mmHg, indicating that patient selection, rather than timing alone, may be another key determinant of outcomes. Moreover, different etiologies of cardiogenic shock involve distinct pathophysiology, and long-term mortality risk remains influenced by factors not directly modifiable by VA-ECMO application.^[^^40^^]^ The absence of detailed data on post-VA-ECMO care, use of other MCS devices, cardiac transplantation, and the duration of renal replacement therapy further limits interpretation of the true association between VA-ECMO initiation timing and long-term mortality.^[^^41^^]^

Although VA-ECMO effectively stabilizes hemodynamics in cardiogenic shock, concerns over complication risks often relegate it to rescue therapy.^[^^42^^]^ In this study, no significant differences in neurological, bleeding, or ischemic complications between early and delayed groups were observed. The results of the ECMO—CS trial are consistent with these observations, showing that the incidence of serious adverse events did not differ significantly between patients regardless of whether they ultimately initiated VA-ECMO.^[^^18^^]^ However, for ischemic complications, the meta-analysis showed substantial heterogeneity, likely reflecting differences in outcome definitions: Choi et al.^[^^21^^]^ and Lee et al.^[^^25^^]^ reported overall limb ischemia, whereas Sundermeyer et al.^[^^26^^]^ focused specifically on access site-related ischemia. Additional factors, such as varying cannulation strategies, monitoring protocols, and patient selection, may further contribute. These findings underscore the need for standardized outcome definitions and cautious interpretation of pooled estimates.

Although no direct evidence links early VA-ECMO initiation to reduced complications, patients frequently present with clinically unstable conditions at delayed initiation. Research data indicate that more than half of the late VA-ECMO group underwent VA-ECMO during cardiopulmonary resuscitation,^[^^21^^]^ a procedure that carries inherent complication risks. Conversely, early VA-ECMO initiation is performed under more stable conditions and better-controlled settings, potentially minimizing complications in emergencies. Additionally, early VA-ECMO initiation may reduce inotrope and vasopressor demand through rapid hemodynamic stabilization, mitigate drug-induced tissue ischemia and neurohormonal activation,^[^^43^^,^^44^^]^ and offset VA-ECMO-associated complications.

In summary, when evaluating appropriate candidates based on the SCAI shock classification, researchers should also take into account comorbidities, including hypoxic brain injury following cardiac arrest, as well as management strategies, including left ventricular unloading and vasoactive agents, in order to achieve an optimal balance between risks and benefits.

### Limitations

First, definitions of early and delayed VA-ECMO initiation were variable, which may introduce bias affecting the findings. Second, available studies rarely report outcomes stratified by SCAI shock classification, limiting understanding of which shock severity groups benefit most from early ECMO intervention. Third, existing evidence derives exclusively from observational studies with inherent residual confounding, and no randomized controlled trials have directly investigated optimal VA-ECMO application. Fourth, long-term follow-up data were limited, with insufficient evidence to analyze the effect of early VA-ECMO initiation on long-term mortality. Last, escalated left ventricular afterload and increased pulmonary edema risk are fundamental limitations of VA-ECMO application,^[^^45^^]^ which may be mitigated by concomitant tMCS.^[^^33^^]^ However, variability in tMCS application between studies may confound the interpretation of optimal VA-ECMO initiation timing effects.

## Conclusions

Observational data from this meta-analysis indicate that early VA-ECMO initiation may be associated with lower short-term mortality in patients with cardiogenic shock, without an apparent increase in neurological, bleeding, or ischemic complications. No reduction in long-term mortality was observed. Given that cardiogenic shock is dynamic and heterogeneous, future studies are needed to investigate the SCAI stages and etiologies of cardiogenic shock for optimal timing of VA-ECMO initiation.

## Acknowledgments

None.

## Funding

This work was supported by the 10.13039/501100001809National Natural Science Foundation of China (Grant Nos. 82072232 and 82572459); the Project of Guangdong-Hong Kong-Macao Greater Bay Area as an International Innovation and Technology Hub (Grant No. 2025A0505010023); the Funding by Science and Technology Projects in Guangzhou (Grant Nos. 2025A03J4248, 2025A03J3472, and 2025A04J3478); the 10.13039/501100012226Fundamental Research Funds for The Central Universities (Grant No. 11625315 and 21624318); the Medical Scientific Research Foundation of Guangdong Province, China (Grant No. A2024458); the 10.13039/501100021171Guangdong Basic and Applied Basic Research Foundation (Grant No. 2024A1515220120). The funding body did not participate in the design of the study, the collection, analysis, and interpretation of data, or in the writing of the manuscript.

## Ethics Statement

Not applicable.

## Conflict of Interest

The authors declare that they have no competing interests.

## Data Availability

The data sets used and analyzed during the current study are available from the corresponding author on reasonable request.

## CRediT authorship contribution statement

**Xiang-kun Yuan:** Writing – review & editing, Writing – original draft, Visualization, Investigation, Formal analysis, Data curation, Conceptualization. **Rui Shi:** Writing – review & editing, Writing – original draft, Visualization, Methodology, Investigation, Formal analysis, Data curation, Conceptualization. **Lu-ming Zhang:** Writing – review & editing, Writing – original draft, Visualization, Methodology, Formal analysis, Conceptualization. **Xiang-jie Duan:** Writing – review & editing, Writing – original draft, Visualization, Formal analysis. **Li-jun Li:** Writing – review & editing, Writing – original draft, Validation, Formal analysis. **Hai-yan Yin:** Writing – review & editing, Validation, Methodology, Conceptualization. **Wan-jie Gu:** Writing – review & editing, Supervision, Resources, Project administration, Data curation. **Xavier Monnet:** Writing – review & editing, Validation, Supervision, Methodology.

## Appendix Supplementary materials

Image, application 1
