# Supplementary material for: Timing of veno-arterial extracorporeal membrane oxygenation in cardiogenic shock: A systematic review and meta-analysis
Source: J Intensive Med. 2026 Feb 19;6(4):396–404. doi: 10.1016/j.jointm.2026.01.005 (PMC13323548; doi:10.1016/j.jointm.2026.01.005)
Supplement: Supplementary file 1 [file mmc1.docx]

**Supplementary material**

| **Table of contents** | **Pages** |
| --- | --- |
| 1. Table S1. Search strategy. | 2 |
| 2. Table S2. Definitions of secondary outcomes. | 3 |
| 3. Table S3. Quality assessments of included studies by Newcastle-Ottawa Scale. | 4 |
| 4. Table S4. Definition of cardiogenic shock with Mixed Etiology. | 5 |
| 5. Table S5. Temporal-definition studies and the applied regrouping for studies with more than two timing categories into early versus delayed VA-ECMO initiation. | 6 |
| 6. Table S6. Definitions and Time Intervals of ECMO Initiation in Included Studies. | 7 |
| 7. Table S7. Reported Lengths of Hospital Stay in Studies with In-Hospital Mortality as an Outcome. | 8 |
| 8. Table S8. Adjusted estimates and their associated variables. | 9 |
| **Figure legends** |  |
| Figure S1. Forest plot for short-term mortality comparing early versus delayed VA-ECMO initiation after exclusion of the largest contributing study. | 10 |
| Figure S2. Subgroup analysis for short-term mortality by VA-ECMO initiation definition (procedural vs. temporal). | 11 |
| Figure S3. Subgroup analysis for short-term mortality by cardiogenic shock etiology (AMI, post-cardiotomy, and mixed etiologies). | 12 |
| Figure S4. Funnel plot and Egger test for short-term mortality. | 13 |

**Table S1 Search strategy**

Searched date: December 11, 2024

| **Source: PubMed** | | |
| --- | --- | --- |
| **Search** | **Query** | **Results** |
| #1 | cardiogenic shock[ti] (Extracorporeal[ti] OR Extra corporeal[ti] OR ECMO[ti] OR ECLS[ti]) (timing[tiab] OR late*[tiab] OR delay*[tiab] OR immediate*[tiab] OR intraoperative*[tiab] OR preoperative*[tiab]) | 137 |
| **Source: Embase** | | |
| **Search** | **Query** | **Results** |
| #1 | 'cardiogenic shock':ti | 9964 |
| #2 | extracorporeal:ti | 37604 |
| #3 | extra corporeal':ti | 1047 |
| #4 | ecmo:ti | 7996 |
| #5 | ecls:ti | 554 |
| #6 | #2 OR #3 OR #4 OR #5 | 44836 |
| #7 | timing:ab,ti | 242843 |
| #8 | late*:ab,ti | 2354238 |
| #9 | delay*:ab,ti | 849082 |
| #10 | immediate*:ab,ti | 672942 |
| #11 | intraoperative*:ab,ti | 293046 |
| #12 | preoperative*:ab,ti | 601091 |
| #13 | #7 OR #8 OR #9 OR #10 OR #11 OR #12 | 4454064 |
| #14 | #1 AND #6 AND #13 | 274 |
| **Source: The Cochrane Library** | | |
| **Search** | **Query** | **Results** |
| #1 | (cardiogenic shock):ti,ab,kw | 1752 |
| #2 | MeSH descriptor: [Shock, Cardiogenic] explode all trees | 504 |
| #3 | #1 OR #2 | 1752 |
| #4 | (Extracorporeal):ti,ab,kw | 6463 |
| #5 | (Extra corporeal):ti,ab,kw | 272 |
| #6 | (ECMO):ti,ab,kw | 1052 |
| #7 | (ECLS):ti,ab,kw | 71 |
| #8 | #4 OR #5 OR #6 OR #7 | 6920 |
| #9 | #3 AND #8 | 185 |

**Table S2 Definitions of secondary outcomes**

| **Neurological complications** | |
| --- | --- |
| **Study** | **Definition** |
| Choi et al. 2020 | Cerebrovascular accidents |
| Huang et al. 2018 | Neurological complications |
| Lee et al. 2021 | Stroke |
| Sundermeyer et al. 2024 | Stroke |
|  |  |
| **Bleeding complications** | |
| **Study** | **Definition** |
| Choi et al. 2020 | Bleeding Academic Research Consortium type 3–5 |
| Huang et al. 2018 | Severe GUSTO bleeding |
| Lee et al. 2021 | ECMO-site bleeding |
| Sundermeyer et al. 2024 | Global Utilization of Streptokinase and Tissue Plasminogen Activator for Occluded Arteries criteria |
| Abbreviations: ECMO, extracorporeal membrane oxygenation; GUSTO, Global Utilization of Streptokinase and Tissue Plasminogen Activator for Occluded Coronary Arteries. | |
|  |  |
| **Ischemia complications** | |
| **Study** | **Definition** |
| Choi et al. 2020 | Limb ischemia |
| Lee et al. 2021 | Limb ischemia |
| Sundermeyer et al. 2024 | Access site-related ischemia |
|  |  |
| **Long-term outcomes** | |
| **Study** | **Definition** |
| Choi et al. 2020 | 1-year mortality |
| Huang et al. 2018 | 2-year mortality |
| Lee et al. 2021 | 1-year mortality |

**Table S3 Quality assessments of included studies by Newcastle-Ottawa Scale**

| **Study** | **Selection** | | | | **Comparability** | **Outcome** | | | **Total** |
| --- | --- | --- | --- | --- | --- | --- | --- | --- | --- |
|  | **Exposed** | **Nonexposed** | **Ascertainment** | **Outcome** |  | **Assessment** | **Length of** | **Adequacy of** |  |
|  | **cohort** | **cohort** | **of exposure** | **of interest** |  | **of outcome** | **follow-up** | **follow-up** | **score** |
| Benseghir et al. 2021 | * | * | * | - | - | * | - | * | 5 |
| Choi et al. 2020 | * | * | * | * | * | * | * | * | 8 |
| Huang et al. 2018 | * | * | * | * | ** | * | * | * | 9 |
| Jentzer et al. 2024 | * | * | * | - | ** | * | - | * | 7 |
| Kim et al. 2021 | * | * | * | * | ** | * | - | * | 8 |
| Lee et al. 2021 | * | * | * | * | ** | * | * | * | 9 |
| Pozzi et al. 2023 | * | * | * | - | - | * | - | * | 5 |
| Sundermeyer et al. 2024 | * | * | * | - | ** | * | - | * | 7 |

| **Table S4 Definition of cardiogenic shock with Mixed Etiology** | | | |
| --- | --- | --- | --- |
| **Study** | **Patients** | **Etiology** | **Definition of cardiogenic shock** |
| Jentzer et al. 2024 [N=8619] | Cardiogenic shock | Mixed | ICD-10: R57.0 ICD-9: 785.1 |
| Lee et al. 2021 [N=362] | Refractory cardiogenic shock | Mixed | The criteria for cardiogenic shock included systolic blood pressure <90 mm Hg for 30 min despite adequate fluid resuscitation or the need for inotropes or vasopressors to maintain systolic blood pressure at ≥ 90 mm Hg |
| Sundermeyer et al. 2024 [N=330] | Cardiogenic shock | Mixed | Incidence of first cardiogenic shock symptoms (e.g. chest pain for patients with ischaemic cardiogenic shock, severe dyspnoea for patients with non-ischaemic cardiogenic shock). |

| **Table S5. Temporal-definition studies and the applied regrouping for studies with more than two timing categories into early versus delayed VA-ECMO initiation.** | | | | | | | | | | |
| --- | --- | --- | --- | --- | --- | --- | --- | --- | --- | --- |
| **Temporal definition study** | **Early group [N=6143]** | | | **Intermediate group [N=328]** | | | | **Delayed group [N=2954]** | | |
|  | **Events** | **Total** | **Time window** | **Events** | **Total** | **Time window** | **Regroup** | **Events** | **Total** | **Time window** |
| Benseghir et al. 2021  [N=114] | 49 | 71 | <3h | 14 | 22 | 3-24h | Early | 16 | 21 | 24-48h |
| Jentzer et al. 2024  [N=8619] | 3038 | 5882 | <24h | - | - | - | - | 1497 | 2737 | 24-96h |
| Lee et al. 2021  [N=362] | 41 | 121 | <0.9h | 51 | 122 | 0.9-2.2h | Early | 56 | 119 | 2.2-24h |
| Sundermeyer et al. 2024  [N=330] | 40 | 69 | <2h | 124 | 184 | 2-12h | Delayed | 50 | 77 | 12-24h |

Abbreviations: VA-ECMO, veno-arterial extracorporeal membrane oxygenation.

| **Table S6 Definitions and Time Intervals of ECMO Initiation in Included Studies** | | | | | | | |
| --- | --- | --- | --- | --- | --- | --- | --- |
| **Study** | **Timing of ECMO initiation** | | | | | | **Data presentation format** |
|  | **Inclusion start point** | **Inclusion cutoff point** | **Early group [N=6945]** | | **Delayed group [N=3506]** | |  |
|  |  |  | **Time window** | **Mean time [min]** | **Time window** | **Mean time [min]** |  |
| Benseghir et al. 2021 [N=114] | Aortic unclamping | At discharge | <=24 hours after aortic unclamping | NA | >24 hours after aortic unclamping | NA | NA |
| Choi et al. 2020 [N=147] | Shock onset | At discharge | Shock-to-ECMO | 83 (33, 158) | Shock-to-ECMO | 90 (25, 418) | Median (interquartile range) |
| Huang et al. 2018 [N=46] | Hospital admission | At discharge | Door-to-ECMO | 63 (54, 137) | Door-to-ECMO | 609 (137, 1986) | Median (interquartile range) |
| Jentzer et al. 2024 [N=8619] | Hospital admission | 96 hours after hospital admission | Hospital admission to ECMO<=24 hours | 480 (180, 900) | Hospital admission to ECMO>24 hours | 2820 (1980, 4080) | Median (interquartile range) |
| Kim et al. 2021 [N=184] | Hospital admission | At discharge | ECMO initiated before PCI during the hospitalization | NA | ECMO initiated after PCI during the hospitalization | NA | NA |
| Lee et al. 2021 [N=362] | Shock onset | 24 hours after shock onset | Shock-to-ECMO | NA | Shock-to-ECMO | NA | NA |
| Pozzi et al. 2023 [N=649] | Hospital admission | 6 days after hospital admission | PCI-to-ECMO | 0 (0) | PCI-to-ECMO | 2448 (1728) | Mean (standard deviation) |
| Sundermeyer et al. 2024 [N=330] | Shock onset | 24 hours after shock onset | Shock-to-ECMO | NA | Shock-to-ECMO | NA | NA |

Abbreviations: ECMO, extracorporeal membrane oxygenation; PCI, percutaneous coronary intervention.

| **Table S7 Reported Lengths of Hospital Stay in Studies with In-Hospital Mortality as an Outcome** | | | | | |
| --- | --- | --- | --- | --- | --- |
| **Study** | **Patients** | **Patient Population** | **Intervention Group** | **Length of Hospital Stay (days)** | **Data Presentation Format** |
| Benseghir et al. 2021 [N=114] | Post-cardiotomy refractory cardiogenic shock | All patients | All patients | 17.8 (18.8) | Mean (standard deviation) |
| Choi et al. 2020 [N=147] | Acute myocardial infarction complicated by refractory cardiogenic shock | Only survived patients | Early | 12.0 (3.5, 56.5) | Median (interquartile range) |
|  |  |  | Delayed | 17.5 (8.0, 42.0) | Median (interquartile range) |
| Jentzer et al. 2024 [N=8619] | Cardiogenic shock | All patients | Early | 13.0 (4.0, 26.0) | Median (interquartile range) |
|  |  |  | Delayed | 16.0 (7.0, 31.0) | Median (interquartile range) |

**Table S8 Adjusted estimates and their associated variables**

| **Study** | **Adjusted estimates** | **Adjusted variables** |
| --- | --- | --- |
| Choi et al. 2020 | In-hospital mortality aOR 0.373 (0.152-0.914) | Age, sex, diabetes mellitus, current smoker, previous history of myocardial infarction, ST-segment elevation myocardial infarction, left ventricular ejection fraction, ENCOURAGE score, culprit lesion location, multi-vessel disease, fluoroscopy guidance, and mechanical ventilation. |
| Kim et al. 2021 | 30-day mortality aOR 0.66 (0.47-0.93) | Pre-PCI ECMO, age, bystander CPR, initial, shockable rhythm, duration of CPR ≥53 minutes (median), shockable rhythm at ER, door-to-balloon time >=113 minutes (median), and successful therapeutic hypothermia |
| Lee et al. 2021 | 30-day mortality aOR 0.56 (0.34-0.91) | History of coronary artery occlusive disease and history of cerebrovascular accident. |
| Jentzer et al. 2024 | In-hospital mortality aOR 0.836 (0.733-0.941) | Age, sex, race, weight, MAP, pH, bicarbonate, preceding arrest, number of vasopressors, and MCS devices. |

Abbreviations: CPR, cardiopulmonary resuscitation; ECMO, extracorporeal membrane oxygenation; ENCOURAGE, prEdictioN of Cardiogenic shock OUtcome foR AMI patients salvaGed by VA-ECMO; ER, emergency room; MAP, mean arterial pressure; MCS, mechanical circulatory support; OR, odds ratio; PCI, percutaneous coronary intervention.

**Figure S1. Forest plot for short-term mortality comparing early versus delayed VA-ECMO initiation after exclusion of the largest contributing study.**


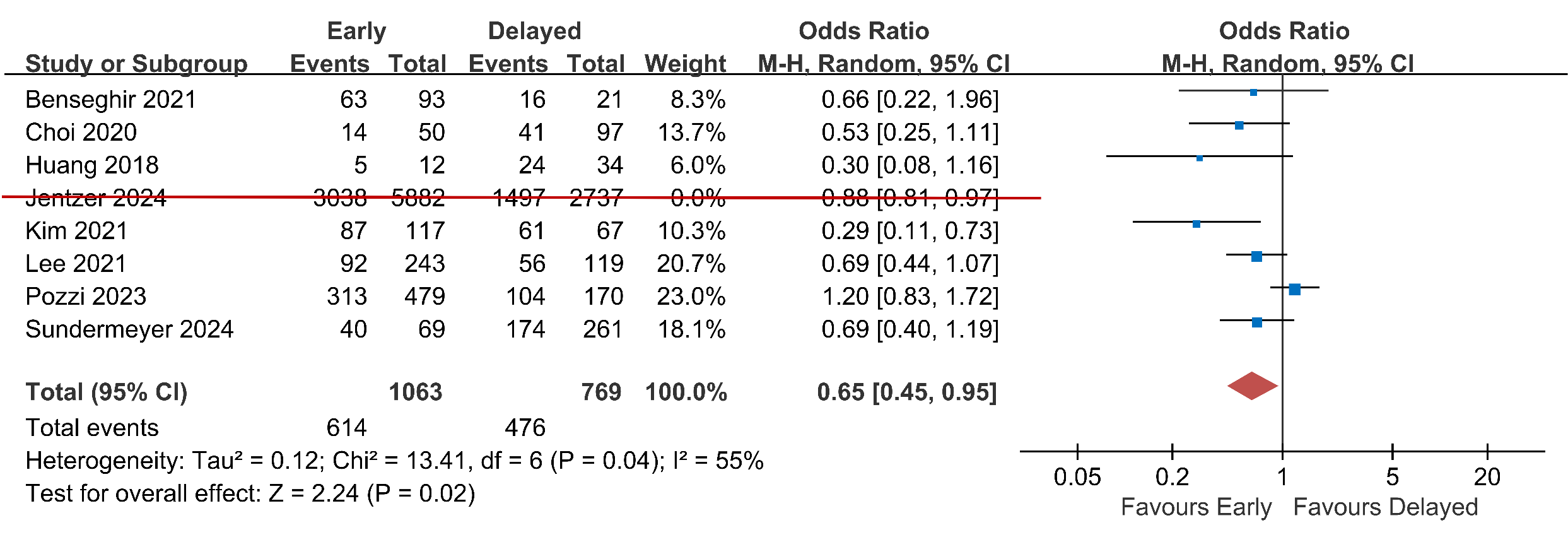


**Figure S2. Subgroup analysis for short-term mortality by VA-ECMO initiation definition (procedural vs. temporal).**


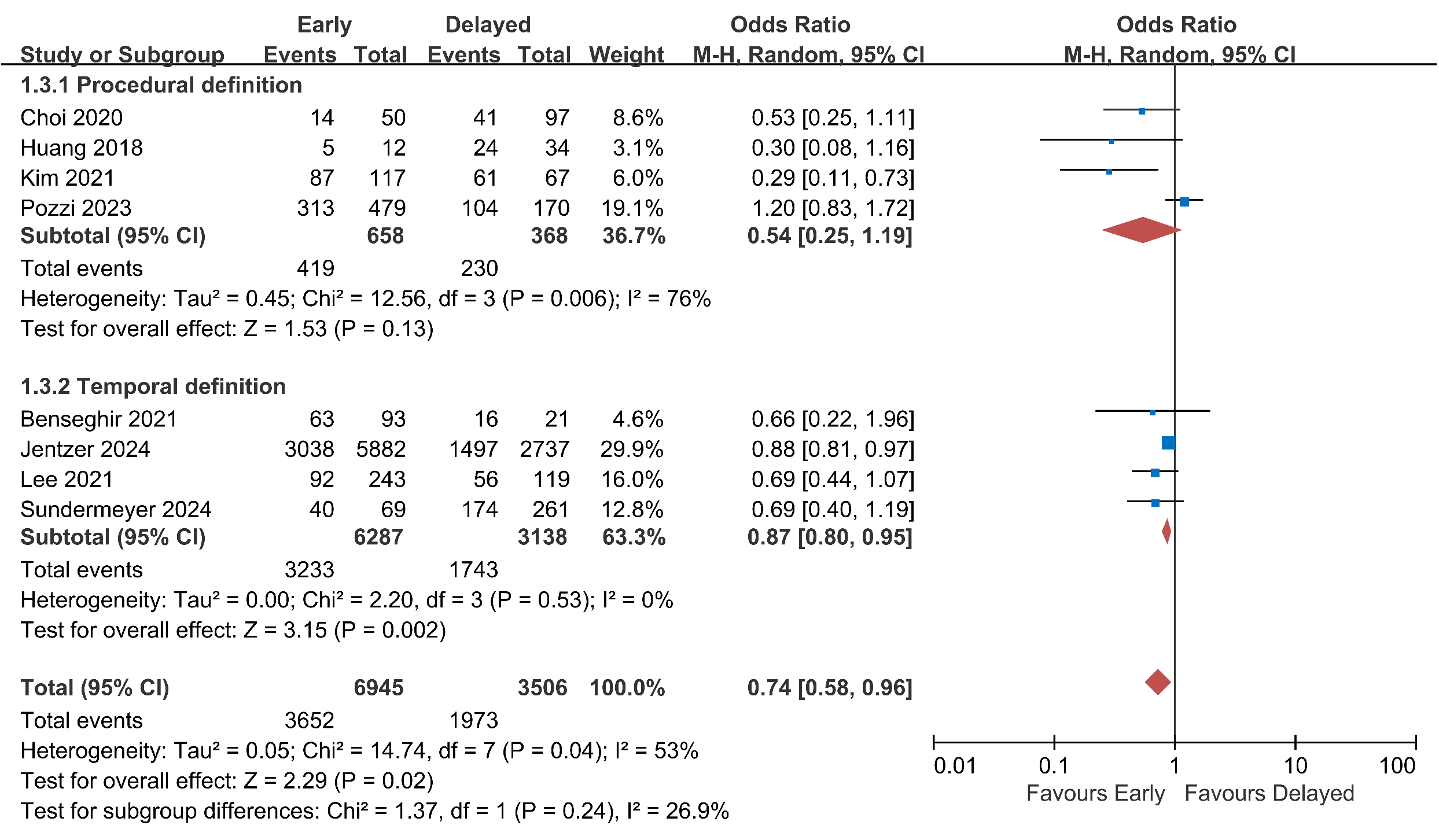


**Figure S3. Subgroup analysis for short-term mortality by cardiogenic shock etiology (AMI, post-cardiotomy, and mixed etiologies).**

**
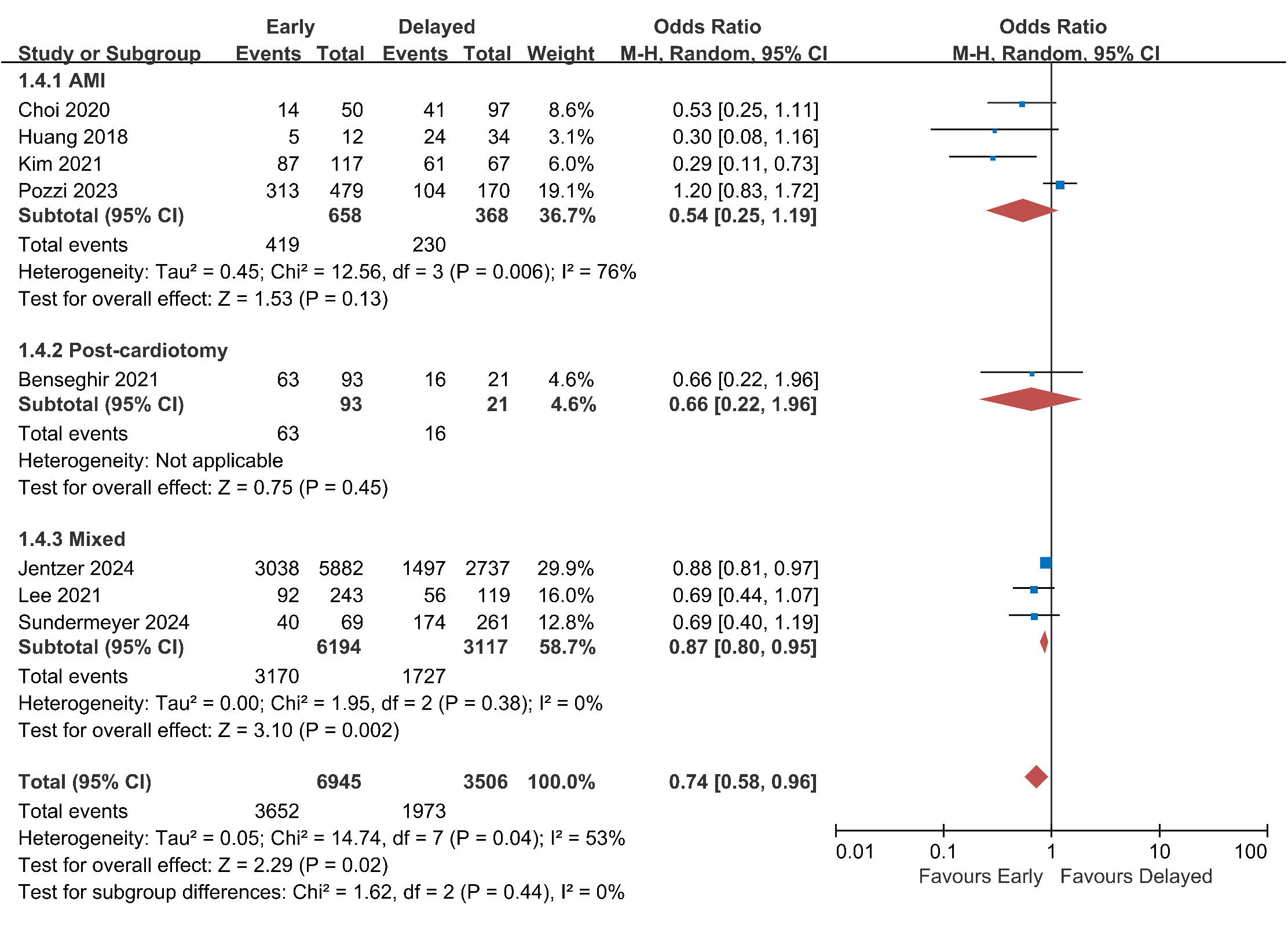
**

**Figure S4. Funnel plot and Egger test for short-term mortality.**

**
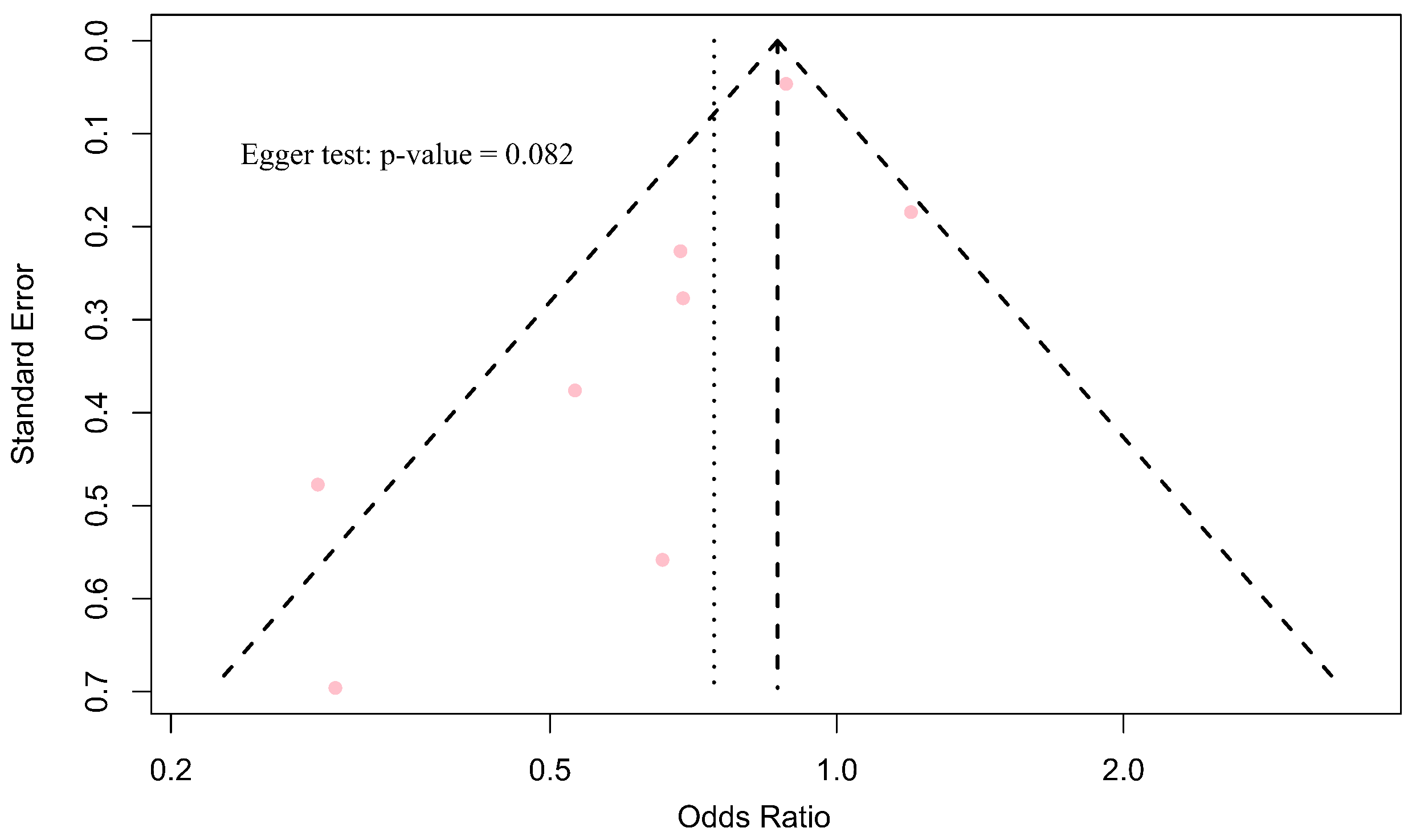
**
